# Astrocytic expression of the Alzheimer’s disease risk allele, ApoEε4, potentiates neuronal tau pathology in multiple preclinical models

**DOI:** 10.1038/s41598-021-82901-1

**Published:** 2021-02-09

**Authors:** Angela Marie Jablonski, Lee Warren, Marija Usenovic, Heather Zhou, Jonathan Sugam, Sophie Parmentier-Batteur, Bhavya Voleti

**Affiliations:** 1 Neuroscience, MRL, Merck & Co., Inc, 770 Sumneytown Pike, West Point, PA 19486 USA; 2 Genetics and Pharmacogenomics, MRL, Merck & Co., Inc, 2000 Galloping Hill Rd, Kenilworth, NJ 07033 USA

**Keywords:** Alzheimer's disease, Neuroscience, Diseases of the nervous system, Molecular neuroscience

## Abstract

ApoEε4 is a major genetic risk factor for Alzheimer’s disease (AD), a disease hallmarked by extracellular amyloid-beta (Aβ) plaques and intracellular neurofibrillary tangles (NFTs). The presence of the ApoEε4 allele is associated with increased Aβ deposition and a role for ApoEε4 in the potentiation of tau pathology has recently emerged. This study focused on comparing the effects of adeno-associated virus (AAV)-mediated overexpression of the three predominant human ApoE isoforms within astrocytes. The isoform-specific effects of human ApoE were evaluated within in vitro models of tau pathology within neuron/astrocyte co-cultures, as well as in a transgenic tau mouse model. Tau aggregation, accumulation, and phosphorylation were measured to determine if the three isoforms of human ApoE had differential effects on tau. Astrocytic overexpression of the human ApoEε4 allele increased phosphorylation and misfolding of overexpressed neuronal tau in multiple models, including the aggregation and accumulation of added tau oligomers, in an isoform-specific manner. The ability of ApoEε4 to increase tau aggregation could be inhibited by an ApoEε4-specific antibody. This study indicates that astrocytic expression of ApoEε4 can potentiate tau aggregation and phosphorylation within neurons and supports a gain of toxic function hypothesis for the effect of hApoEε4 on tau.

## Introduction

Alzheimer’s disease (AD) represents the most common form of dementia in older adults, estimated to make up 60–80% of cases^1^. Clinically, AD is characterized by both cognitive and non-cognitive impairments including progressive loss of memory, deficits in problem solving, language impediments, and neuropsychiatric symptoms^2^. These clinical manifestations present alongside histological alterations in the brain including atrophy, synaptic loss, the appearance of extracellular senile plaques composed of amyloid-beta (Aβ), and the formation of intracellular neurofibrillary tangles (NFTs) comprised of the protein tau^3^. Most cases of AD occur later in life after the age of 65, known as late-onset AD (LOAD)^4^.

The human ε4 allele of Apolipoprotein E (*APOEε4*) is expressed in more than half of AD patients and *APOEε4* remains the greatest genetic risk factor to date for LOAD, making it an important therapeutic target^5,6^. Carrying one *APOEε4* allele increases the risk of LOAD about three-fold and carrying two alleles increases the risk roughly 12-fold^7,8^. Nearly fourteen percent of the population are estimated to carry at least one copy of *APOEε4* making it an imperative subject of study in terms of how it can contribute to AD pathobiology^9^.

ApoE is a 299 amino acid protein which plays a key role in lipid metabolism and is primarily expressed peripherally in the liver by hepatocytes and macrophages^10^. In the central nervous system (CNS), ApoE is primarily produced and secreted by astrocytes followed by oligodendrocytes, microglia, and ependymal layer cells. ApoE can also be expressed by neurons under pathological conditions where it plays a major role in the redistribution of cholesterol and phospholipids for cellular repair and remodeling^11,12^. Researchers have demonstrated that stress can robustly increase *APOE* mRNA levels within neurons, suggesting that increased ApoE expression by neurons may be neuroprotective^13^. In humans, the APOE gene is polymorphic with three high frequency alleles: *APOEε2*, *APOEε3* and *APOEε4*^14^. The *APOEε3* allele is the most prevalent whereas *APOEε2* and *APOEε4* are associated with beneficial and detrimental AD risks respectively. These three isoforms differ at only two key residues at Cys-112 and Arg-158 which lead to profound structure and function changes for the ApoE protein^15^. The residues are found in the N-terminal region of the protein near the LDL-receptor binding domain^16^ and their mutations result in differential binding to lipoproteins and some lipoprotein receptors^17^.

The protein encoded by the *APOEε4* allele is immunoreactive in both amyloid plaques and NFTs which define the AD phenotype, suggesting that it may play a role in both hallmarks of the disease^18^. Multiple pathological, biomarker, and clinical studies have shown that cerebral Aβ accumulation is positively correlated with the *APOEε4* in cognitively normal subjects, mild cognitive impairment (MCI) cases, and symptomatic AD patients^19–24^^,^^25^. In addition to Aβ, studies have also suggested a link between ApoE and tau. CSF ApoE levels strongly correlate with CSF Tau/pTau in *APOEε4*-carriers^26^ and multiple preclinical cellular studies have demonstrated the effects of ApoEε4 and its fragments on the formation of intracellular NFT-like inclusions^27,28^. Other in vivo studies have identified the ApoEε4 protein as a modifier of tau pathology and neurodegeneration in transgenic tau mouse models^29,30^ and in human iPSC-derived neurons^31^. In ApoE targeted-replacement mice, increased tau phosphorylation was detected in ApoEε4 animals by one month of age alongside learning impairments^32^. Human ApoEε4 cerebral organoids derived from iPSC-derived cell types also exhibit increased hyperphosphorylation of tau^33^.

Very recently, a single case report described a *PSEN1* carrier with a rare *APOE* mutation at R136 known as the Christchurch mutation. This patient presented with minimal cognitive decline and reduced NFTs despite high Aβ burden suggestive of a protective effect of the mutation. This mutation disrupted the protein’s ability to bind heparin, potentially affecting its binding to lipoprotein receptors and other proteoglycans, which have been reported to be involved in tau uptake and spread^34,35^. Another recent study demonstrated that LRP1, an ApoE receptor, was required for the uptake of tau in human iPSC neurons^36^, further suggesting a potential role for ApoE receptor binding in tau pathology.

These studies suggest that ApoEε4 may play a role in tau pathology, which would be critical as findings from clinical PET, CSF, and post-mortem studies suggest that the pathological aggregation of tau is closely linked to patterns of neurodegeneration and cognitive deficits in AD^37–39^. Still, several uncertainties remain, including whether ApoEε4 overexpression in astrocytes alone is sufficient to drive changes in tau. The goal of this study was to examine the role of the three human ApoE (hApoE) isoforms, specifically when produced by astrocytes, which are a predominant source of ApoE within the CNS. Our data indicate that astrocytic ApoEε4 converts neuronal tau to a more pathological state both in vitro and in vivo. This phenomenon was not observed with the other ApoE isoforms and was present in both tau overexpression and tau oligomer models. This makes the role for astrocytic ApoE in tau pathogenesis of key interest to identify new possible strategies for targeting ApoE in AD.

## Results

### Targeting human ApoE isoform expression to the astrocytes of rat hippocampal co-cultures

We set out to establish if there were any pathological effects of the astrocytic human ApoE isoforms (hApoEε2, hApoEε3, and hApoEε4) on neuronal tau. To do this, neuron and astrocyte co-cultures were prepared from E18 rat hippocampi. Using the co-culture preparation protocol described in Methods, these co-cultures routinely produce co-cultures with roughly ~ 60% neurons and ~ 40% astrocytes (Supplementary Fig. S1). To target hApoE isoforms specifically in astrocytes, AAV9 vectors were used to express the flag-tagged hApoE isoforms under the control of the astrocyte-specific promoter, glial fibrillary acidic protein (GFAP). The GFAP promoter has widely been used to direct expression specifically in astrocytes in vitro and in vivo^42^. The C-terminal flag-tag on the hApoE protein was used to differentiate the hApoE constructs from the rodent ApoE still present in these models. To test these constructs, equivalent amounts of AAV9 (each containing one of the three P_GFAP_::hApoE constructs) were added to rat hippocampal co-cultures at day in vitro (DIV) 5 at a multiplicity of infection (MOI) of 20,000 GC/cell. To determine if this level of hApoE overexpression was toxic to the cells, we assessed the viability of co-cultures five days following AAV transduction of the three hApoE isoforms compared to a control P_GFAP_::EV group using high-content imaging. Astrocyte numbers were counted as the number of GFAP-positive nuclei and were similar among EV (1244 ± 91.02), hApoEε2 (1131 ± 169.3), hApoEε3 (1107 ± 236.4), and hApoEε4 (973.5 ± 176.3) groups (data represented as mean ± SEM) (*p* = 0.8809, one-way ANOVA). Neuronal numbers were counted as the number of MAP2-positive nuclei and were also similar among EV (1024 ± 69.02), hApoEε2 (905.8 ± 179.4), hApoEε3 (797.8 ± 138.7), and hApoEε4 (714.5 ± 39.05) groups (data represented as mean ± SEM) (*p* = 0.3350, one-way ANOVA). We did observe, however, that there was a non-significant trend for increasing neuronal loss in an ε4 > ε3 > ε2 manner (Supplementary Fig. S1). By imaging the flag tag on the hApoE construct in combination with neuronal and astrocytic markers, we also confirmed overexpression of hApoE within GFAP-labeled astrocytes. However, flag staining could also be detected extracellularly, although no strong co-localization between flag and MAP2-labeled neurons was observed (Supplementary Fig. S1). To test if this staining pattern may have been due to secretion of hApoE by astrocytes, we confirmed that there was no extracellular staining of a GFP construct alone under the control of synapsin- or GFAP-driven promoters. In that experiment, GFP was found exclusively in MAP2-labeled neurons and GFAP-labeled astrocytes using synapsin and GFAP promoters respectively (Supplementary Fig. S1).

**Figure 1:**
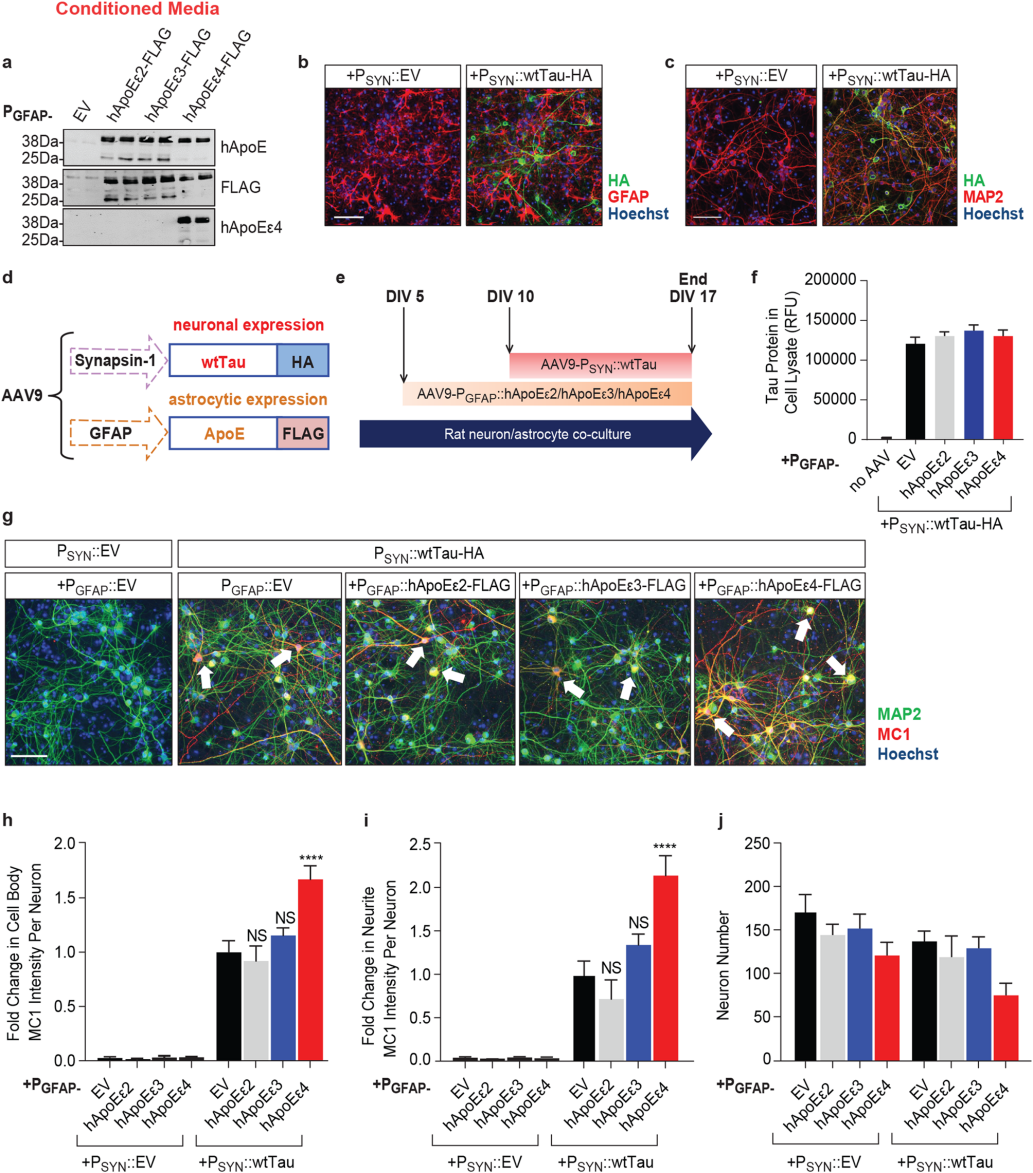
Astrocytic hApoEε4 increases neuronal tau MC1 pathology without altering tau expression levels. Scale bar of representative images represents 50 µM. High-content imaging and its associated quantification represents data from one independent experiment. **(a)** Representative Western blot to detect released hApoE in the clarified conditioned media of co-cultures five days after transduction with P_GFAP_:: EV/hApoEε2/hApoEε3/hApoEε4 (*n* = 2 technical replicates/group). Full Western blot image can be found in Fig. S4 of Supplementary Information. **(b)** Immunocytochemistry to detect tau-HA in rodent co-cultures seven days after being transduced with P_SYN_::EV or P_SYN_::wtTau (DIV17). Cultures were co-stained with GFAP to label astrocytes and Hoechst to label nuclei. **(c)** Immunocytochemistry to detect tau-HA in rodent co-cultures seven days after being transduced with P_SYN_::EV or P_SYN_::wtTau (DIV17). Cultures were co-stained with MAP2 to label neurons and Hoechst to label nuclei. **(d)** Description of AAV vectors and constructs to express wtTau in neurons and hApoE in astrocytes. **(e)** Schematic illustrating the experimental paradigm to overexpress human ApoE in astrocytes beginning at DIV5, followed by the overexpression of wild type human tau in neurons beginning at DIV10 before experiment end at DIV17. **(f)** Quantification by AlphaLISA of HA-tagged human tau expression in cell lysates from co-cultures seven days after transduction with tau (DIV17). (*n* = 3 sample means/group). **(g)** Immunocytochemistry to detect MC1-positive tau in neurons (MAP2-positive cells) of co-cultures at DIV17. Hoechst stain was used to label nuclei. Arrow heads point to representative MC1-positive cell bodies and neurites. **(h,i)** Quantification of MC1 intensity in MAP2-labeled cell bodies **(h)** and neurites **(i)** (*n* = 6 wells/group). MC1 data were normalized to the P_GFAP_::EV + P_SYN_::wtTau group and presented as the fold change. **(j)** Quantification of MAP2-positive Hoechst-stained nuclei by high-content imaging (*n* = 6 wells/group). NS—not significant, *p < 0.05, ****p < 0.001, Tukey multiple comparisons test following one-way ANOVA.

As mentioned above, the staining pattern we observed for ApoE (Supplementary Fig. S1) is likely a result ApoE’s typical expression and secretion patterns: ApoE is released from astrocytes following its production and processing, where it can later be taken up by neurons to transport lipids in a partially isoform-specific manner^43^. Using Western blot, we confirmed the secretion of full-length flag-tagged hApoE (~ 38 kDa) among all three hApoE isoform groups in the clarified conditioned media of co-cultures five days following transduction with hApoE (Fig. 1a). This was expected based on ApoE’s known production and subsequent release from astrocytes. As depicted in the representative Western blot (Fig. 1a), we also observed a reduction in hApoEε4 protein abundance in the conditioned media across several experiments. This is consistent with studies using in vivo hApoE models, which have reported a decrease in hApoEε4 abundance compared to hApoEε2 and hApoEε3 within the plasma, brain and cerebrospinal fluid^44^. The reduced protein abundance of hApoEε4 in this model also correlated with a reduction in hApoE fragment production (< 38 kDa) compared to hApoEε2 and hApoEε3. We specifically noticed the absence of a 25 kDa fragment in the hApoEε4 group. A 25 kDa fragment of hApoE has also been reported missing in AD patients carrying the *APOEε4* allele compared to healthy controls^45^.

### Co-expression of neuronal tau and astrocytic hApoEε4 increases pathological tau in neurons

Wild type (WT) human 4R2N tau (wtTau) with a C-terminal HA tag was virally delivered into neurons using AAV9 with a synapsin (SYN) promoter. Seven days following P_SYN_::EV or P_SYN_::wtTau-HA transduction, cells were fixed and stained with a HA antibody to stain for AAV-driven tau and either MAP2 to label neurons or GFAP to label astrocytes. We observed no colocalization between HA and GFAP (Fig. 1b), but robust co-localization between HA and MAP2 (Fig. 1c). These data suggest that the AAV-mediated overexpression of tau is limited to neurons as expected (Fig. 1b,c).

To assess the effect of astrocytic hApoE isoforms on neuronal tau, tau was transduced five days after the transduction of the three hApoE isoforms (hApoEε2, hApoEε3, or hApoEε4) in astrocytes (at DIV10) (Fig. 1d,e). Five days following hApoE transduction was chosen for optimal expression of hApoE prior to introducing human tau into neurons. An AlphaLISA was used to measure total human tau which detected no changes in human tau protein levels when tau was co-overexpressed with the three hApoE isoforms, even when compared to the P_GFAP_::EV control (*p* = 0.49, one-way ANOVA) (Fig. 1f).

To assess tau pathology in this system, co-cultures overexpressing human tau in the presence of the three human ApoE isoforms were fixed seven days after tau transduction (Fig. 1e). High-content imaging analysis was performed to quantify cell viability and conformational changes of tau within MAP2-labeled neurons detected by the MC1 antibody (Fig. 1g), which detects a pathological conformation of tau species in the brains of AD patients, but not in healthy controls^46^. In the MC1-detected conformation, the N-terminus of tau interacts with its C-terminal third microtubule-binding repeat domain, resulting in a partially folded pathological structure detectable in the early stages of AD^46^. No pathological MC1-positive tau could be detected in co-cultures transduced with P_SYN_::EV, whereas neuronal tau overexpression led to a robust accumulation of MC1-posiitve tau (Fig. 1g–i) in both neuronal cell bodies and neurites. When astrocytes were co-transduced with the hApoE isoforms, only hApoEε4 significantly potentiated MC1 levels. Transduction of hApoEε4 led to a 1.5-fold increase of MC1 intensity in MAP2-labeled cell bodies over EV, whereas the other hApoE isoforms had no effect on MC1 (*p* < 0.0001, one-way ANOVA) (Fig. 1h). In addition, hApoEε4 also resulted in a twofold increase of MC1 intensity in MAP2-labeled neurites over EV control, whereas the expression of the other hApoE isoforms had no effect (Fig. 1i) (*p* < 0.0001, one-way ANOVA). These data suggest that the presence of the hApoEε4 isoform in astrocytes leads to an increase in a pathological confirmation of tau within neurons without changing tau protein level.

We also monitored the effects on cellular viability and found that tau expression itself did not significantly alter the number of neurons. However, co-expression of tau and hApoEε4 led to a non-significant 50% decrease in neuron number compared to the EV control group in the presence of tau (*p* = 0.0665, one-way ANOVA) (Fig. 1j). This result could reflect the impact on neuronal viability by increased tau pathology in the presence of hApoEε4.

We confirmed that co-expression of hApoEε4 with tau did not significantly change expression of the tau-HA construct. No changes in neuronal HA intensity was found among hApoE groups co-transduced with the HA-tagged tau construct by immunocytochemistry (Supplementary Fig. S2). There was also no increase in the expression of the transduced tau construct (4R-tau) or the endogenous rodent tau (predominantly 3R-tau) in the hApoEε4 group detected by Western blot (Supplementary Fig. S2). Furthermore, no changes in any oligomeric tau species^47^ or tau cleavage products^48^ were detected in those cellular lysates in the presence of hApoEε4 to explain the observed increase in MC1 (Supplementary Fig. S2). These additional findings suggest that hApoEε4 is not increasing MC1-positive tau through increasing expression of the tau construct. These findings are consistent with our measurement of human tau expression seen in Fig. 1f. In that experiment, no change in the expression of the human tau construct was detected with co-expression of various hApoE isoforms. However, it is still important to acknowledge that not all tau-HA-expressing cells develop MC1 pathology (Supplementary Fig. S3). Further work is required to understand why only some neurons developed MC1 pathology in this model.

### Astrocytic hApoEε4 leads to hyperphosphorylation of neuronal tau in an epitope-specific manner

**Figure 2:**
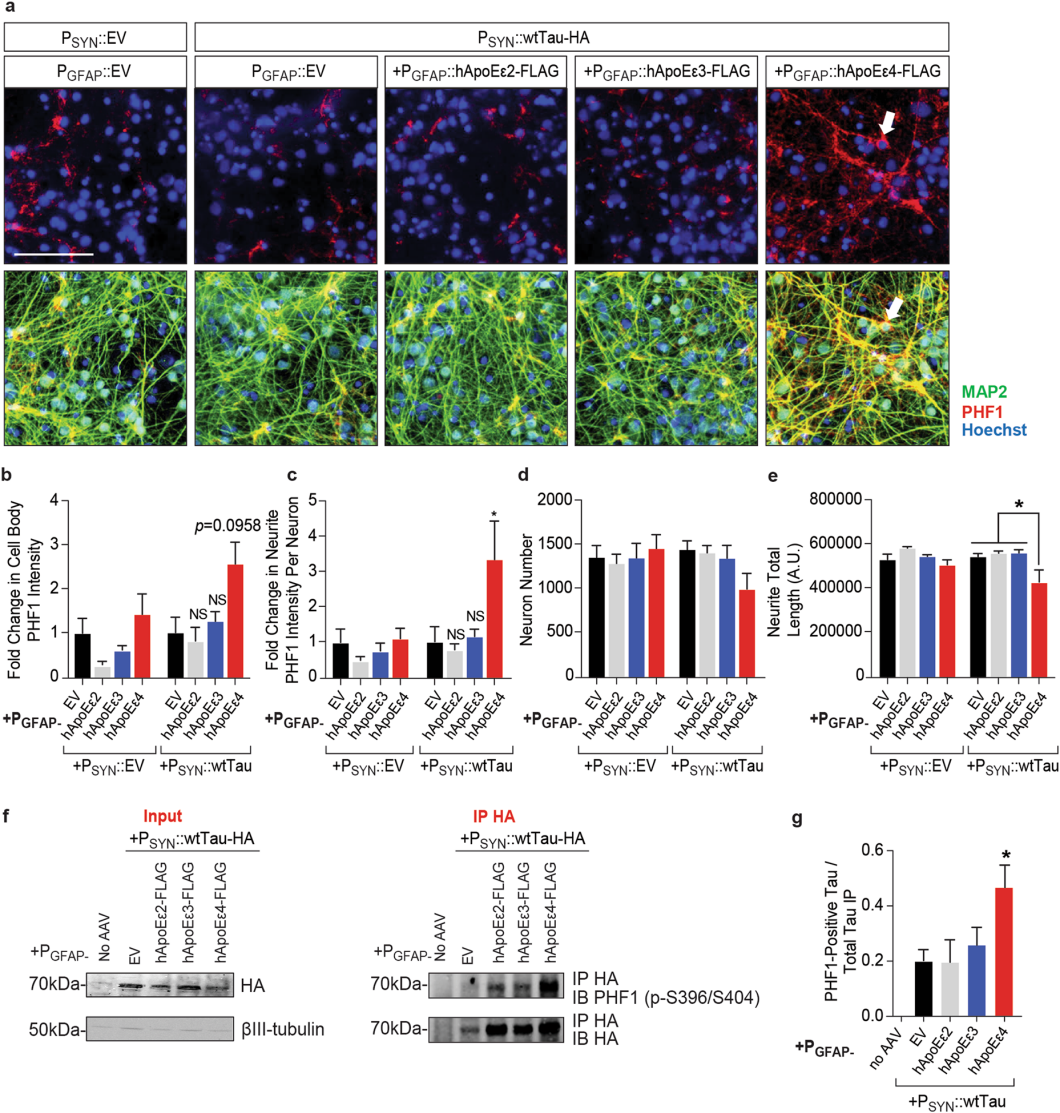
Astrocytic hApoEε4 increases phosphorylation of tau within neurons. Scale bar of representative images represents 25 µM. High-content imaging and its associated quantification represents data from one independent experiment. **(a)** Immunocytochemistry of phosphorylated tau (PHF1) in co-cultures seven days after neuronal transduction with EV control or human tau (DIV17). AAV transduction was completed as described in **(a)**. Cells were stained for PHF1 to visualize tau phosphorylation and MAP2 to label neurons. Hoechst stain was used to label nuclei. Arrow head points to PHF1-positive cell body and associated neurites in the P_SYN_::wtTau + P_GFAP_::hApoEε4 condition. Top panel depicts images without MAP2 imaging overlay to aid in visualization. **(b,c)** Quantification of PHF1 staining intensity in MAP2-labeled cell bodies (B) and neurites **(d)** (*n* = 4–6 wells/group). Data were normalized to the P_GFAP_::EV + P_SYN_::wtTau group. *NS* not significant, *p < 0.05, Tukey multiple comparisons test following one-way ANOVA. **(e)** High-content imaging to measure MAP2-positive Hoechst-labeled nuclei (*n* = 4–6 wells/group). **(f)** Quantification of the total neurite length of processes extending from MAP2-positive cell bodies (*n* = 4–6 wells/group). *p < 0.05, Tukey’s multiple comparisons test following one-way ANOVA. **(g)** Representative Western blots of immunoprecipitated HA-tagged tau (~ 70 kDa) from co-culture systems seven days after transduction with tau (DIV17) (IP HA) and the cell lysates (input) used to perform the immunoprecipitation (IP HA). HA and PHF1 antibodies were used to detect immunoprecipitated HA-tagged tau and pTau respectively. Full Western blot image can be found in Fig. S4 of Supplementary Information. **(h)** Quantification of band densities of Western blot depicted in panel **(h)** (*n* = 3). Data were normalized to the quantity of HA-tagged tau immunoprecipitated. *p < 0.05, Kruskal–Wallis test.

Tau hyperphosphorylation is one of the main drivers of conformational changes in tau^49^. To determine if hApoEε4 overexpression also impacted the phosphorylation of neuronal tau, tau phosphorylation was evaluated by high-content imaging and biochemical methods. With the same experimental paradigm as described above (Fig. 1d), co-cultures overexpressing hApoE isoforms and tau were fixed and stained with the PHF1 antibody which detects tau phosphorylated at S396/S404 (Fig. 2a).

P_SYN_::wtTau overexpression in the presence of the P_GFAP_::EV control did not increase PHF1 staining compared to its P_SYN_::EV control. However, hApoEε4 expression led to 2.5- and threefold increases in PHF1intensity within MAP2-labeled cell bodies (*p* = 0.0081, one-way ANOVA) (Fig. 2b) and neurites (*p* = 0.0046, one-way ANOVA) (Fig. 2c) respectively when combined with tau overexpression. Using the MAP2-positive cells mask for high-content imaging analysis, we again observed a non-significant trend for hApoEε4 to exacerbate neuronal loss in the presence of tau overexpression (*p* = 0.5372, one-way ANOVA) (Fig. 2d). This reduction of neuronal viability was further associated with a significant ~ 25% reduction in neurite outgrowth over EV control which was not present with the other hApoE isoforms (*p* = 0.0011, one-way ANOVA) (Fig. 2e). These data suggest that hApoEε4 has a negative effect on overall neuronal health when combined with tau overexpression.

To further confirm this effect observed by high-content imaging, we utilized the HA-tag on transduced tau to immunoprecipitate human tau and measured the abundance of phosphorylation on that fraction by Western blot. Consistent with the high-content imaging studies, an increase in phosphorylation at the PHF1 site (pS396/pS404) was observed in hApoEε4-expressing co-cultures alone over the EV control group (Fig. 2f). We noticed that there tended to be more tau-HA immunoprecipitated among the hApoE groups compared to empty vector control in these experiments. However, as previously described (Supplementary Fig. S2), we did not observe any increase in tau-HA expression in the presence of hApoE overexpression in the cellular lysates which were used for the immunoprecipitation (Fig. 2f). When controlling for the quantity of tau-HA immunoprecipitated, there was a two-fold increase in phosphorylation at the PHF1 (pS396/pS404) site following tau co-expression with hApoEε4 (*p* = 0.0015, Kruskal–Wallis test) (Fig. 2g). These findings agree with other studies that have suggested hApoEε4 can drive tau phosphorylation^45^. Taken together with our MC1 findings, these studies confirm that astrocytic hApoEε4 can contribute to both tau phosphorylation and misfolding without the necessity of driving expression of hApoEε4 in neurons and showed an isoform-specific effect unique to hApoEε4.

### Astrocytic hApoE leads to oligomerization of neuronal tau in an isoform-specific manner

**Figure 3:**
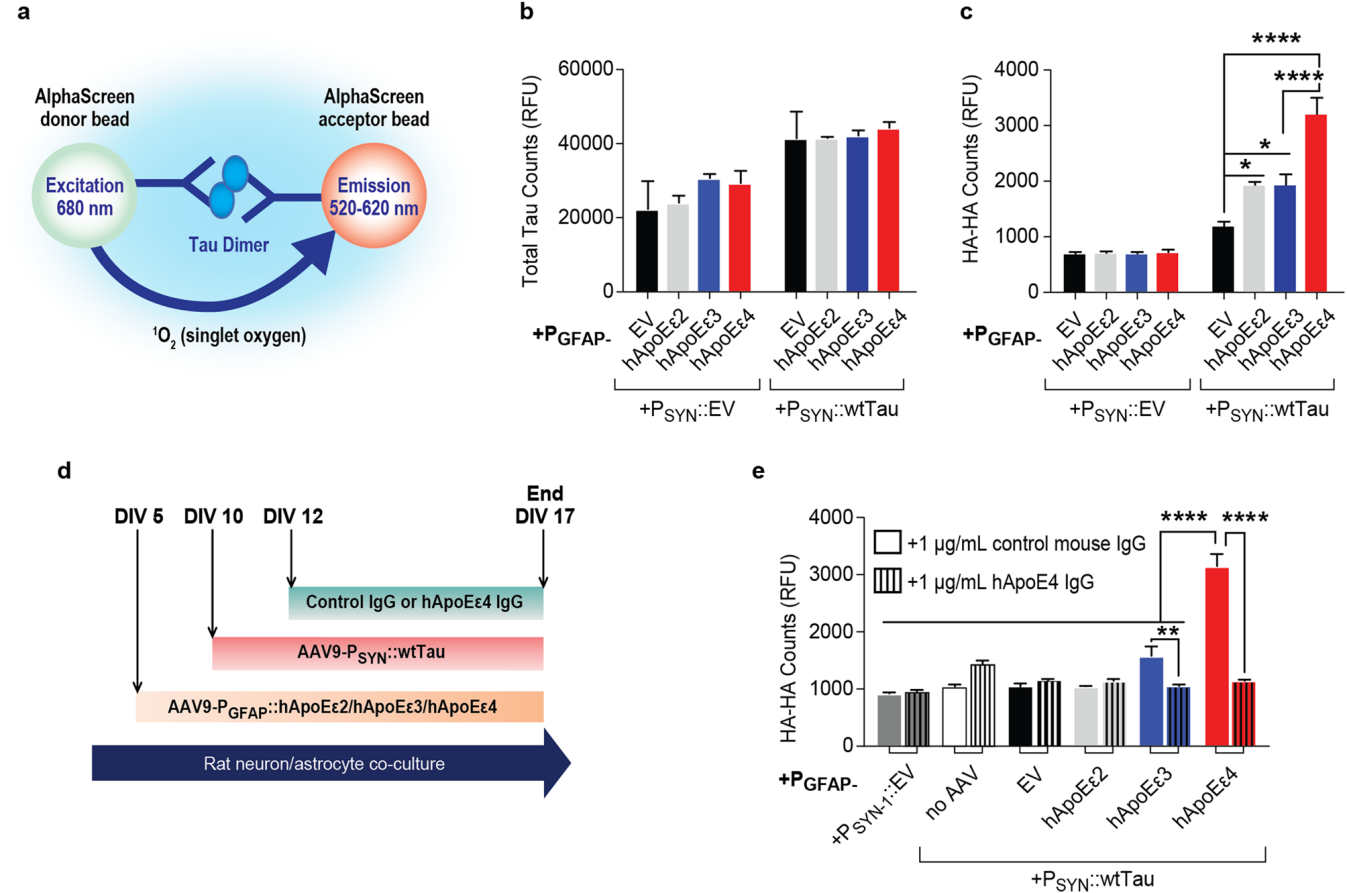
hApoEε4 increases the oligomerization of neuronal tau. Quantification represents data from one independent experiment. **(a)** Schematic to illustrate the AlphaLISA principle for detecting the oligomerization of HA-tagged tau constructs using HA-HA AlphaLISA. Image was created using Adobe Illustrator CC2018 v.22.0.0 (https://www.adobe.com/Adobe/Illustrator). **(b)** AlphaLISA results to quantify the abundance of total (rat + human) tau in co-cultures 7 days after transduction with tau (DIV17) (*n* = 3 sample means/group). **(c)** Results of HA-HA AlphaLISA seven days after transduction with tau (DIV17) (*n* = 7 sample means/group). *p < 0.05, ****p < 0.001, Tukey’s multiple comparisons test following one-way ANOVA. **(d)** Experimental paradigm to treat AAV-P_GFAP_::hApoE + AAV-P_SYN_::wtTau co-cultures with relevant IgG’s. Schematic was created using Microsoft Word v2002 (https://www.microsoft.com/en-us/microsoft-365/word). **(e)** Results of HA-HA AlphaLISA seven days after transduction with HA-tagged tau following treatment with 1 μg/mL control or hApoEε4-specific antibodies as described in panel **(b)** (*n* = 3 sample means/group). **p < 0.01, ****p < 0.0001, Tukey’s multiple comparisons test following one-way ANOVA.

The MC1 conformation of tau is detected in both soluble forms of tau and in paired helical filaments (PHFs)^50–52^. Tau misfolding and hyperphosphorylation also leads to tau oligomerization and aggregation before the formation of higher order species of tau aggregates that make up insoluble NFTs^53^. We used an HA-HA bead-based AlphaLISA to quantify oligomeric species of tau (Fig. 3a). When one HA tag is in close enough proximity to another separate HA tag, excitation permits the emission of a signal which can be measured to allow for the quantification of the dimerization and oligomerization of tau. The rationale behind this assay is that one HA-tagged tau molecule contains only one epitope to which the HA antibody could bind. Thus, one tau molecule could not interact with both the HA-bound acceptor and donor beads simultaneously to generate an AlphaLISA signal. However, oligomeric tau molecules in close enough proximity could generate an AlphaLISA signal by providing multiple epitopes for the HA antibody to bind. Other groups have already taken advantage of this system and published assays for similar oligomerizing proteins, such as for alpha-synuclein^54^.

To confirm any changes in tau oligomerization were not due to changes in tau expression, we first utilized a total tau AlphaLISA using tau epitopes capable of detecting both rodent and human tau to measure total tau among all groups. We detected a roughly two-fold overexpression of tau protein in P_SYN_::wtTau groups over P_SYN_::EV groups. As described earlier, this two-fold increase in total tau was unaffected by hApoE isoform overexpression (*p* = 0.9432, one-way ANOVA), confirming that hApoE overexpression did not affect tau expression in our model. Human ApoE overexpression also had no influence on rodent tau expression in the P_SYN_::EV cohort (*p* = 0.4857, one-way ANOVA) (Fig. 3b). Astrocytic hApoEε4 expression in co-cultures, however, led to a 50% increase in the HA-HA AlphaLISA signal over hApoEε2 and hApoEε3 co-cultures within the P_SYN_::Tau cohort, suggesting that hApoEε4 potentiates tau oligomerization (*p* < 0.0001, one-way ANOVA). (Fig. 3c). These data indicate that astrocytic hApoEε4 can shift neuronally-expressed tau to a more oligomeric and aggregated state and this effect appears to be independent of any change in tau expression levels.

### Anti-hApoEε4 antibody reduces tau oligomerization induced in hApoEε4-containing co-cultures

Recent work has identified hApoE antibodies as one viable therapeutic approach to reduce the pathological effects of hApoEε4, such as reducing amyloid beta plaque load and memory loss^55–57^. For example, HJ6.3, an antibody that targets endogenous murine ApoE, suppressed Aβ pathology in APPswe/PS1Δ9 mice^55^. We wanted to determine if an hApoEε4-specific antibody could reduce the observed increased tau oligomerization by hApoEε4 in this co-culture system.

We tested the efficacy of an hApoEε4 (9D11, Biolegend) antibody to reduce tau oligomerization measured by the HA-HA AlphaLISA. For this experiment, the treatment paradigm is outlined by the schematic in Fig. 3d. When cultures were treated with a control antibody, we detected a nearly twofold increase in the HA-HA signal by hApoEε4 compared to all other groups (*p* < 0.0001, one-way ANOVA). However, when co-cultures were treated with the hApoEε4 antibody, the HA-HA signal was reduced three-fold back to control levels when compared to cultures which were treated with a control antibody (*p* < 0.0001, one-way ANOVA) (Fig. 3e). We noted that there was no longer a significant increase in the HA-HA signal by hApoEε2 or hApoEε3 over empty vector when co-cultures were treated with the control antibody alone in this experiment (Fig. 3e) as previously observed in Fig. 3c. We believe that this could be due to some form of signal interference by the antibody treatment itself. Treatment with the hApoEε4 antibody similarly reduced the HA-HA signal by approximately 50% in hApoEε3 co-cultures (*p* < 0.0001, one-way ANOVA) (Fig. 3e). This may have also been a result of some non-specific cross-reactivity between the hApoEε3 protein and the hApoEε4 antibody which was below the level detectable by our previous Western blot (Fig. 1a).

### hApoEε4 increases the intracellular accumulation of cholesterol and tau oligomers

**Figure 4:**
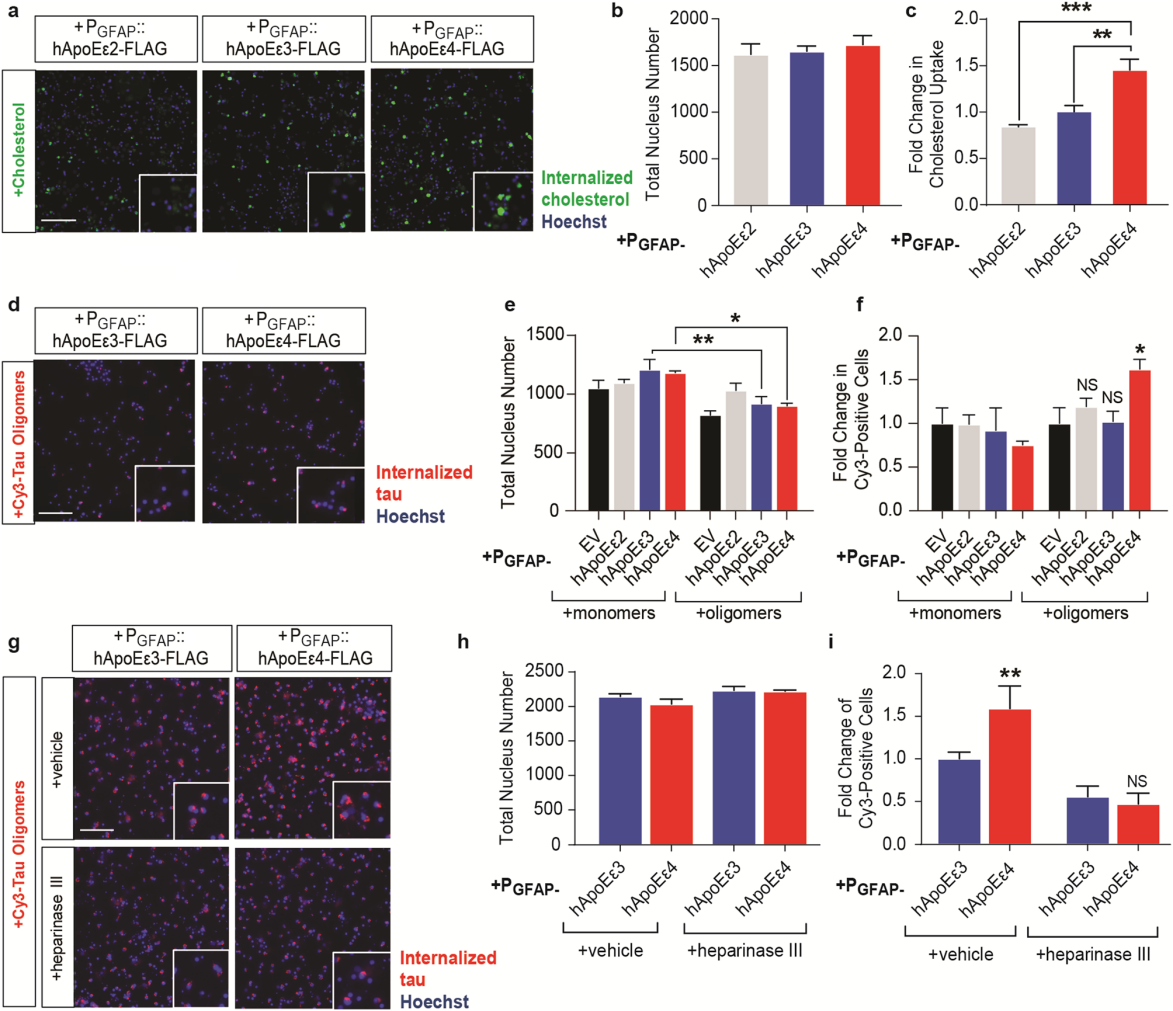
Astrocytic hApoEε4 alters the dynamics of cholesterol and tau oligomer accumulation. Scale bar of representative images represents 50 µM. High-content imaging and its associated quantification represents data from one independent experiment. **(a)** Representative live-cell imaging of GFP-labeled cholesterol internalization one day following a 4-h incubation in co-cultures. Hoechst stain was used to visualize nuclei. **(b)** Quantification of Hoechst-labeled nuclei (*n* = 6 wells/group) in panel **(a)**. **(c)** Quantification of the total GFP (cholesterol) intensity in the cytoplasm of cells by high-content image analysis. Data are depicted as fold change over P_GFAP_::hApoEε3 group (*n* = 6 wells/group). **p < 0.01, ***p < 0.005, Tukey’s multiple comparisons test following one-way ANOVA. (d) Representative live-cell imaging of cy3-tagged tau internalization 24 h following cy3-tau addition. Hoechst stain was used to visualize nuclei. **(e)** Quantification of Hoechst-labeled nuclei by high-content imaging (*n* = 5 wells/group). *p < 0.05, **p < 0.01, Tukey’s multiple comparisons test following one-way ANOVA. **(f)** Quantification of the number of cy3-positive cells. Data are depicted as the fold changes over respective monomer or oligomer-treated P_GFAP_::EV groups (*n* = 5 wells/group). *NS* not significant, *p < 0.05, Tukey’s multiple comparisons test following one-way ANOVA. **(g)** Representative live-cell imaging of cy3-tagged tau internalization 24 h following tau addition. Cells were pre-treated with vehicle or 150 mU heparinase III for four hours prior to cy3-tau oligomer addition. Hoechst stain was used to visualize nuclei. **(h)** Quantification of Hoechst-labeled nuclei by high-content imaging (*n* = 3 wells/group). **(i)** Quantification of the number of cy3-positive cells. Data are depicted as the fold change over P_GFAP_::hApoEε3 + vehicle group (*n* = 3 wells/group). *NS* not significant, **p < 0.01, Fisher’s LSD test following one-way ANOVA.

Previous reports have shown that hApoEε4 status influences changes in the trafficking, uptake, and clearance of lipoproteins between neurons and astrocytes^58^. To assess dysregulation of cholesterol homeostasis in this AAV-P_GFAP_::hApoE model, we treated co-cultures with a fluorescently labeled cholesterol molecule (NBD cholesterol) and washed the cells four hours later. This cholesterol molecule contains an environment-sensitive probe which emits a signal when localized in the membrane's interior to monitor cholesterol trafficking. One day after the addition of NBD cholesterol, cells were imaged to determine cholesterol uptake and accumulation within cells (Fig. 4a). These experiments were further performed in the presence of a green background suppressor dye in the media to prevent any extracellular fluorescence^40^. High-content imaging detected no changes in cell viability with these treatments (*p* = 0.7212, one-way ANOVA) (Fig. 4b), but detected a 50% increase in intracellular cholesterol levels remaining one day after cholesterol addition in hApoEε4 co-cultures compared to hApoEε2 and hApoEε3 co-cultures (*p* = 0.0002, one-way ANOVA) (Fig. 4c).

Given the change in the uptake and/or clearance of cholesterol molecules within cells, it was not known whether hApoEε4 may also alter the dynamics of uptake and/or clearance of tau. Clinical evidence shows that tau pathology may spread through axonally connected brain regions^59^. Based on the prion hypothesis of tau transmission, our laboratory previously established a model where internalized tau oligomers were shown to cause tau pathology in human iPSC-derived neurons inducing endogenous tau phosphorylation and aggregation without requiring tau overexpression or a tau mutation^40^. These oligomer preparations were previously characterized and found to contain tau monomers, dimers, trimers, and tetramers^40^. Interestingly, the LRP1 receptor, an ApoE receptor, was recently identified as a mediator of monomeric and oligomeric tau uptake in cells^36^. Thus, we next asked if hApoEε4 also affected the uptake and/or accumulation of tau in cells by treating cells with cy3-tagged tau monomers or oligomers five days after hApoE transduction. Tau was washed from the cells after a four-hour incubation period and cells were imaged one day later to measure internalized tau uptake assessing both tau uptake and accumulation (Fig. 4d). The experiment was completed in the presence of a red background suppressor dye in the media to inhibit extracellular fluorescence which could be due to residual cell-bound tau remaining after wash steps^40^. Treatment with tau oligomers led to a modest reduction in cell viability across all groups but was only significant when comparing monomer to oligomer treatments within hApoEε3 and hApoEε4 groups (*p* < 0.0001, one-way ANOVA) (Fig. 4e). We observed that hApoEε4 led to a 50% increase in the intracellular accumulation of cy3-tau oligomers over EV control, whereas hApoEε2 and hApoEε3 exerted no effect on tau accumulation (*p* = 0.0131, one-way ANOVA) (Fig. 4f).

Heparin sulfate proteoglycans have been extensively characterized as a major mediator of tau uptake^35,60^. Interestingly, the same domain of hApoE which binds to LDL receptors, such as LRP1, also binds to heparin and thereby HSPGs. As mentioned earlier, the *APOE* Christchurch mutation appeared to be protective against tau pathology in a case report and that mutation also severely disrupted the binding of hApoE to heparin^34^. Given these connections between tau spread, ApoE, and HSPGs, we asked if HSPG’s might mediate the increased accumulation of tau observed under the hApoEε4 condition. We pre-treated cells with vehicle or heparinase III, which removes heparan sulfate (HS) polysaccharides on the cell surface to impair HSPG’s^35,40^. Heparinase treatment has been used extensively in the past to impair HSPG’s and study the role of HSPG’s on tau uptake^35,40,61^. Our group previously published that pre-treatment with heparinase III successfully reduced heparan sulfates in human iPSC neuronal cultures to impair tau uptake^40^. Unfortunately, the methods used to detect this change in cell-surface heparan sulfates were not compatible with the rodent co-cultures used in this study. Still, following the pre-treatment with vehicle or heparinase, we added cy3-tagged tau oligomers as previously described in Fig. 4d. We found that heparinase treatment had no effect on cell viability (*p* = 0.0771, one-way ANOVA) (Fig. 4g,h), but led to a 50% impairment of tau oligomer uptake in general and further reversed the increased accumulation of tau oligomers caused by hApoEε4 (*p* = 0.0048, one-way ANOVA) (Fig. 4g,i). These data suggest that hApoEε4 and its binding partners may regulate the uptake and/or accumulation of tau within cells, assuming that the heparinase treatment was successful as we have observed in other cell types^40^.

### hApoEε4 exacerbates tau pathology independently of tau overexpression

**Figure 5:**
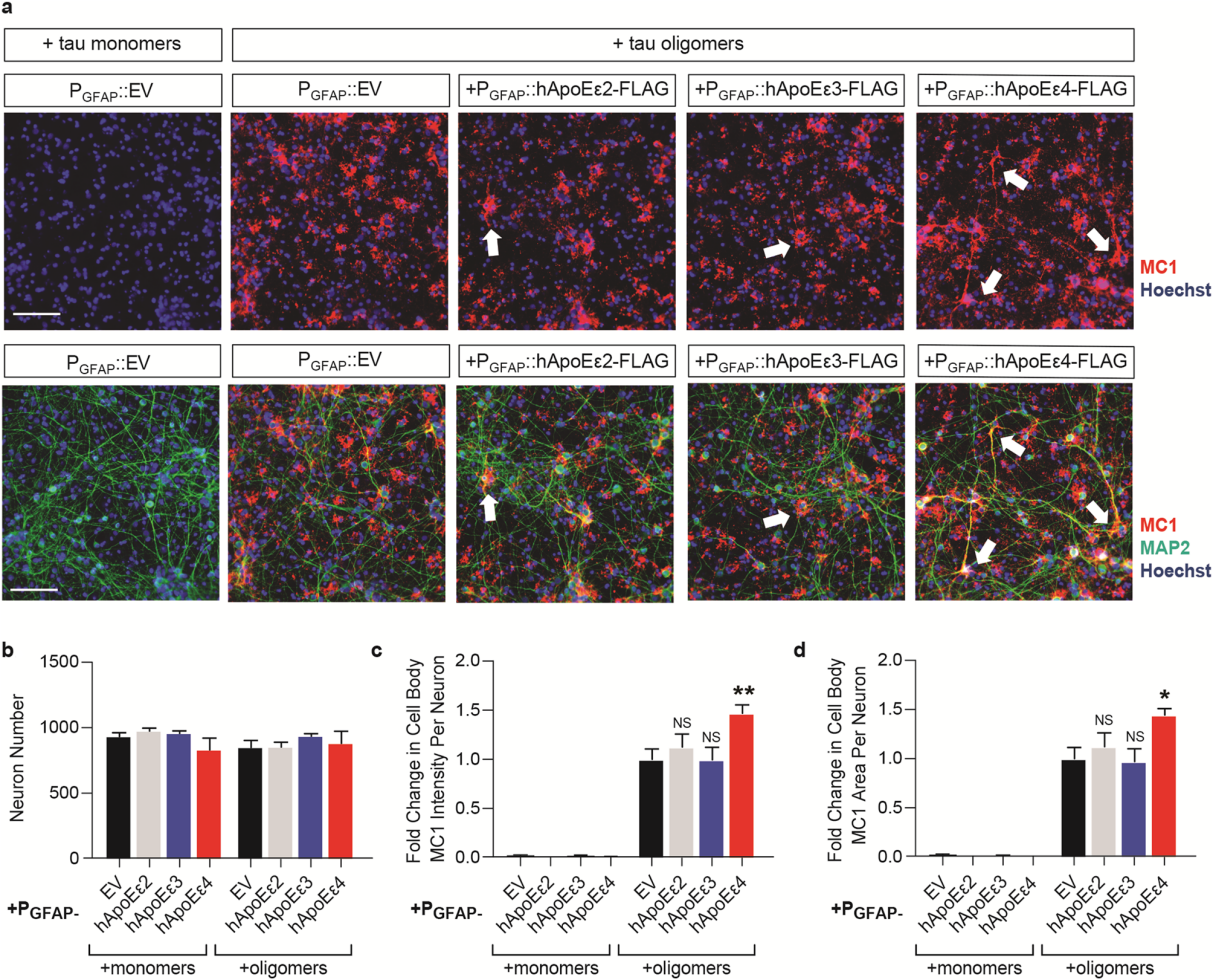
Astrocytic hApoEε4 potentiates MC1 pathology produced by tau oligomers. Scale bar of representative images represents 50 µM. High-content imaging and associated quantification represents data from one independent experiment. **(a)** Immunocytochemistry of co-cultures five days after addition with 100 nM of tau monomers or tau oligomers. Oligomers were added five days after transduction with the P_GFAP_ construct of interest. Cells were fixed and stained with MC1 to measure tau pathology and MAP2 to label neurons. Hoechst stain was used to label nuclei. Arrowheads highlight examples of MC1-positive cell bodies and neurites. Top panel depicts images without MAP2 imaging overlay to aid in visualization. **(b)** High-content imaging to quantify MAP2-positive Hoechst-labeled nuclei (*n* = 4 wells/group). **(c,d)** Quantification of MC1 intensity **(c)** and MC1 area **(d)** in MAP2-labeled cell bodies (*n* = 4 wells/group). Data were normalized to the oligomer-treated P_GFAP_::EV group (*n* = 4 wells/group). *NS* not significant, *p < 0.05, **p < 0.01, Tukey multiple comparisons test following one-way ANOVA.

Since overexpression of hApoEε4 increased the accumulation of tau oligomers, we next determined if these changes in tau accumulation by hApoEε4 affected subsequent tau pathology. Human ApoE-expressing co-cultures were treated with untagged tau oligomers 5 days after viral transduction of the hApoE isoforms. After incubation for one day, cells were washed multiple times and co-cultures were fixed and stained for MAP2 and MC1 five days after tau oligomer addition. No MC1 could be detected in co-cultures treated with monomeric tau, whereas treatment with the same concentration of oligomeric tau led to widespread accumulation of MC1 pathology (Fig. 5a). There were no changes in neuron viability (*p* = 0.4461, one-way ANOVA) (Fig. 5b) among groups. MC1 pathology induced by the treatment with tau oligomers was significantly enhanced by 50% in the condition of astrocytic hApoEε4 expression compared to EV control when MC1 intensity (*p* < 0.0001, one-way ANOVA) (Fig. 5c) and MC1 area (*p* < 0.0001, one-way ANOVA) (Fig. 5d) were quantified. In contrast, expression of hApoEε2 and hApoEε3 had no effect on MC1 staining following the addition of tau oligomers. This demonstrates that hApoEε4 is capable of increasing tau pathology independently of tau overexpression and may contribute to the spread and accumulation of pathological tau in the brain.

### Increased ApoEε4 expression results in increased tau pathology and phosphorylation in a Tau P301L mouse model

To explore the in vivo translatability of these findings, we targeted the expression of hApoE isoforms using the AAV-P_GFAP_ delivery system to the dorsal hippocampus of rTg(tauP301L)4510 (WT:Car) mice. These heterozygous WT:Car tauP301L transgenic mice express a ~ 3-fold overexpression of mutated human tau^41^ and do not show any significant tau pathology, signs of neurodegeneration, or behavioral deficits during 8–16 weeks of age, which were the ages used in this study. This is in opposition to homozygous Car:Car rTg(tauP301L)4510 mice with at least 13-fold increases in tau expression levels.

**Figure 6:**
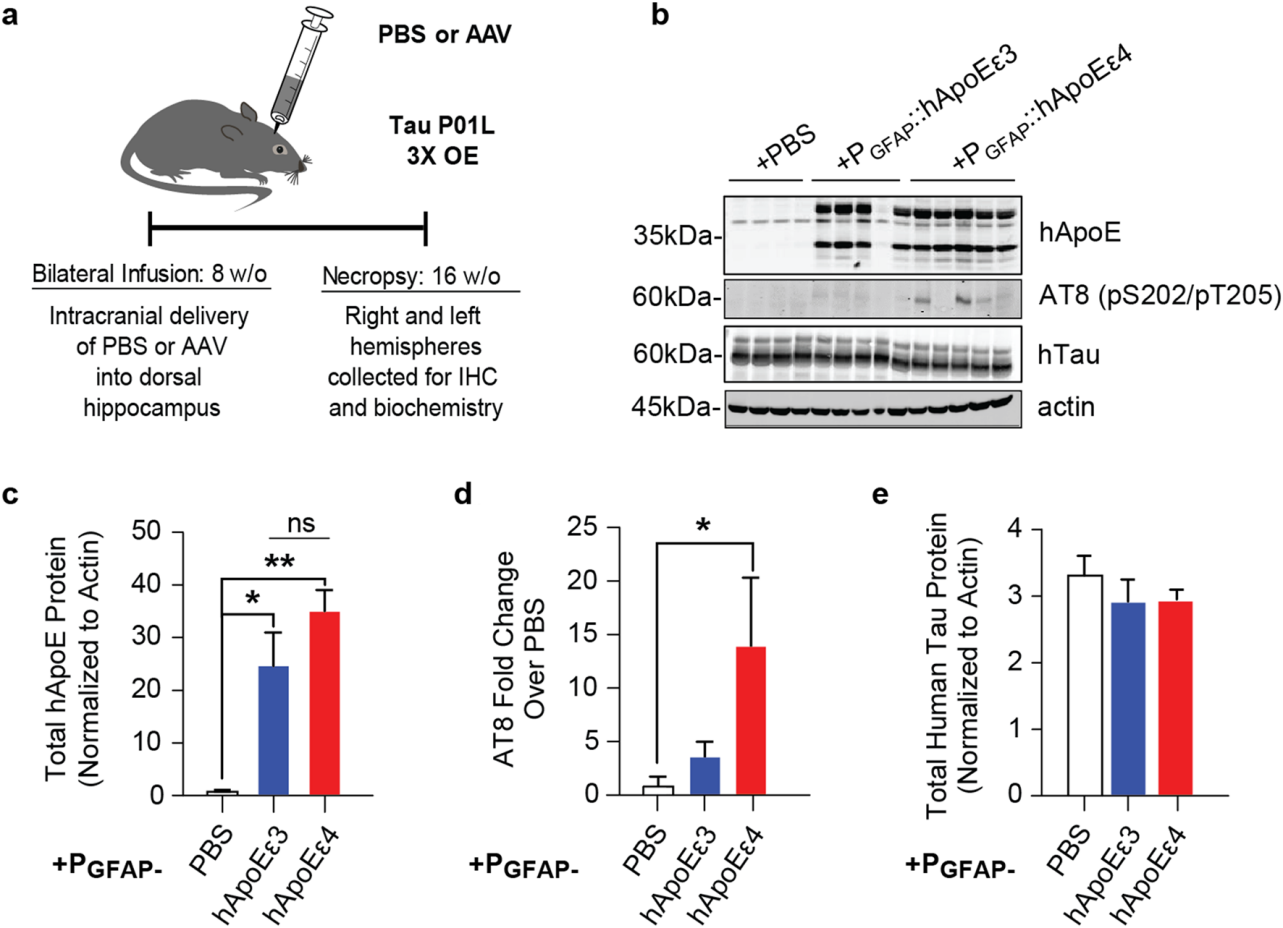
hApoEε4 expression in the hippocampus of 3× Tau P301L mice leads to increased tau phosphorylation in vivo. **(a)** Schematic of experimental design. Image was created using Adobe Illustrator CC2018 v.22.0.0 (https://www.adobe.com/Adobe/Illustrator). **(b)** Representative Western blot of total lysates from hippocampal tissues from hippocampus-injected heterozygous tauP301L mice to depict hApoE, AT8 (tau-pS202/pS205), and total tau levels among groups. Actin was used a loading control. Full Western blot image can be found in Fig. S4 of Supplementary Information. **(c)** Densiometric quantification of hApoE bands normalized to loading control from Western blot depicted in panel **(b)** (*n* = 4–5 animals/group). *NS* not significant, *p < 0.05, **p < 0.01, Tukey’s multiple comparisons test following one-way ANOVA. **(d)** Quantification of band densities (*n* = 4–5 animals/group) of Western blot depicted in panel b for AT8. *p < 0.05, Fisher’s LSD test following one-way ANOVA. Data were normalized to loading control and are depicted as fold change over PBS-sham animals. **(e)** Densiometric quantification of human tau bands normalized to loading control from Western blot depicted in panel **(b)** (*n* = 4–5 animals/group).

Mice were infused with PBS or the AAV of interest in the dorsal hippocampus at eight weeks of age and euthanized eight weeks later (16 weeks of age) to evaluate both expression of the hApoE isoforms and tau phosphorylation (Fig. 6a). A robust comparable overexpression of ApoE in both hApoEε3- and hApoEε4- (*p* = 0.3141, Tukey’s multiple comparison’s test following one-way ANOVA) injected animals was confirmed after the respective intra-hippocampal injection of the hApoE isoforms over PBS control (*p* = 0.0017, one-way ANOVA) (Fig. 6b,c). Western blot analysis of the hippocampal homogenates from tau transgenic mice injected with hApoEε4 indicated a significant > 10-fold increase in the tau phosphorylation marker AT8 (pS202/pT205) in comparison to mice injected with PBS (*p* = 0.0540, one-way ANOVA) (Fig. 6b,d). Importantly, there was no difference in the level of total tau expression among any of the groups (*p* = 0.5680, one-way ANOVA) (Fig. 6b,e). This finding confirms that hApoEε4 is capable of increasing tau phosphorylation an in vivo context. Further extensive evaluations of this model will be the focus of another separate study.

## Discussion

This work provides new evidence for a role of hApoEε4 in the potentiation of tau phosphorylation and aggregation. These findings were isoform-specific and unique to the ε4 isoform of hApoE and were not observed under hApoEε2 or hApoEε3 conditions. Astrocytic overexpression of hApoEε4 enhanced tau pathology in multiple models, including tau overexpression and tau oligomers models in vitro and the heterozygous tauP301L mouse model. Given the strong correlation between tau spread in the brain and cognitive decline^3^, this role of hApoEε4 in tau pathology could explain the significantly higher rates of clinical disease progression among *APOEε*4-carriers^30,62–64^.

Past studies have similarly shown that hApoEε4 can increase tau phosphorylation and induce cognitive deficits in vivo but have often used neuronal overexpression of hApoEε4^29,65–67^. Some other studies have also reported that hApoEε4 does not affect tau pathology^68^. These conflicting findings are likely due to the type of hApoE used, the lipidation status of hApoE, and how hApoE (or tau) was expressed in each model system. Differences in hApoE expression patterns are critical because hApoE isoforms are differentially lipidated following their release from astrocytes, with hApoEε4 being hypolipidated compared to the other hApoE isoforms^69–71^. Overexpressed hApoE was previously shown capable of being lipidated^17,72^ and lipoproteins from cultured astrocytes of hApoEε4-targeted replacement mice were deficient in promoting neurite outgrowth, suggestive of a functional consequence of lipidation^72^.Thus, we chose to use AAV-mediated overexpression to drive expression of the hApoE isoforms from astrocytes. However, although our model has the advantage of selective expression of hApoE expression in astrocytes, one limitation is the continued presence of endogenous rodent ApoE. It is possible that this rodent ApoE could play a role in our observed effects when the hApoE isoforms are overexpressed, but this seems unlikely since tau pathology was only exacerbated in the astrocytic hApoEε4 condition, and not under hApoEε2 or hApoEε3 conditions. Another potential limitation of this study is the use of an overexpression paradigm to introduce hApoE in the model. Human ApoE knock-in models^30^ which drive hApoE expression using the endogenous APOE promoter can be advantageous to further perform mechanistic validation experiments.

One of the remaining questions from our study is whether the lipidation status on hApoE could drive these observed allelic differences. Secreted hApoE was not produced in the amount needed from the co-cultures in this study to quantitatively assess changes in lipidation. We did, however, detect an increased accumulation of exogenously applied cholesterol in hApoEε4 conditions compared to the other hApoE isoforms. This is consistent with other models utilizing glia established from hApoEε4 iPSC’s lacking any hApoE overexpression, which displayed increased cholesterol accumulation^33^ associated with decreased cholesterol efflux^73^. It would potentially be interesting to determine if this type of lipid dysregulation within hApoEε4 conditions dives the observed tau phenotypes.

Hypolipidation conferred by the hApoEε4 mutation has also been hypothesized to contribute to the decreased stability and fragmentation of the hApoEε4 protein^15,74^. Consistent with this, we observed lower amounts of hApoEε4 in the conditioned media of co-cultures compared to the other hApoE isoforms. However, we did not detect any hApoEε4-specific fragments in the conditioned media of our AAV co-culture model. Specific C-terminal hApoEε4 fragments generated by neurons^29^ have been shown to negatively interact with mitochondria leading to neurotoxicity^75^. In vivo expression of C-terminal hApoEε4 fragments in mice leads to death by 2–4 months of age alongside the presentation of AD-like features of neurodegeneration including phosphorylated tau throughout the cortex and hippocampus^68^. C-terminal fragments of hApoEε4 also accumulate in AD brains within NFTs^27^. Although we did not observe any strong co-localization between the flag-tagged hApoE constructs and MAP2-labeled neurons (Supplementary Fig. S1), we cannot exclude the possibility of neuronal uptake of overexpressed hApoEε4 and further processing within neurons where tau pathology was observed.

It is also possible that released hApoE in our model could bind to its neuronal receptors to affect tau pathology. ApoE is known to bind to heparin sulfate proteoglycans (HSPGs) to clear remnant lipoproteins^71,76^ and HSPGs have also been widely described as a mediator in tau uptake^35,61^. The ApoE receptor, LRP1, was also recently characterized as a mediator of tau uptake^36^. Of note, a recent case study further described the *APOE* R136S Christchurch mutation which led to a significant reduction in tau pathology within the brain of a *PSEN1* carrier and the authors provided evidence that this mutation impaired the binding of ApoE to heparin^34^. Thus, due to the increased evidence for the pathogenic role of trans-synaptic uptake and spread of oligomeric tau species in AD^40,77^, we also tested if astrocytic expression of hApoE isoforms could differentially alter tau endpoints in a tau oligomer model. We found that hApoEε4 enhanced both the accumulation of internalized tau and the subsequent MC1-positive tau pathology following the addition of tau oligomers. Treatment with heparinase III, a common method to impair HSPGs^40^, further blocked the increased accumulation of tau observed in the hApoEε4 condition. Although we were unable to confirm that heparinase III treatment reduced heparan sulfates in our model due to technical limitations, this finding dictates the need to further understand the potential links between ApoE, HSPG’s, and tau. This experiment did not rely on any AAV-mediated tau overexpression, but these methods are still unable to discriminate whether these findings are mediated by an alteration in tau uptake, tau clearance, or both. Multiple studies suggest hApoEε4 can inhibit the clearance of tau, although no changes in tau expression itself could be detected in this model to explain our findings (Supplementary Fig. S2). For example, Chu et al*.* demonstrated that the high-temperature requirement serine peptidase A1 (HtrA1) preferred hApoEε4 over hApoEε3, leading to less HtrA1 being available to clear tau in hApoEε4 condition^78^. Also, hApoEε4 was previously shown to directly inhibit autophagy through competition between hApoEε4 and the autophagy activating protein, TFEB^79^. Interestingly, multiple TFEB-regulated mRNA transcripts critical for autophagy are downregulated in AD *APOEε4*-carrier brains over AD *APOEε3*-carrier brains^79^.

The effects of hApoEε4 on tau pathology described by this study were importantly not limited to these tau in vitro model systems. Astrocytic expression of hApoEε4 in tauP301L heterozygous mice enhanced the phosphorylation of tau. These results differ from others reporting that astrocytic expression alone of hApoEε4 in vivo is not sufficient to drive tau hyperphosphorylation in wild type mice^80^. The low level of mutated tau combined with astrocytic expression of hApoEε4 in our study may have been sufficient to trigger the significant changes in tau phosphorylation that we observed. In other conflicting studies, subtle changes were used in the methods to express hApoE. For example, using different promoters, such as the mouse APOE^30^ versus CBA^81^ promoters, researchers have described different hApoE expression patterns between neurons and glia which could potentially account for these differential outcomes on tau^30,81^. Importantly, similarly to our findings, when more robust hApoE expression could be detected in glia, an increase in tau pathology was observed under hApoEε4 conditions, in conjunction with enhanced neurodegeneration and hippocampal atrophy^30^. That study^30^ further found that these effects on tau could be mediated by differential patterns of microglial activation and neuroinflammation among the hApoE isoforms^30^. New work and more advanced 3D cellular models encompassing microglia are required to better elucidate these mechanisms within the context of our own findings. Although these heterozygous tauP301L mice do not exhibit any behavioral or pathological deficits on their own at the ages studied here, much further work is required to extensively characterize these animals and determine the full spectrum of effects caused by hApoEε4 introduction into the hippocampus. For example, hApoEε4 targeted-replacement mice have been previously found to have deficits associated with various memory tasks^82^, and it would be interesting to know if hApoEε4 introduction as we described could replicate some of those past behavioral findings. Thus, extensive further characterization of the AAV-hApoE in vivo model introduced here will be the subject of a future study.

Although the mechanism underlying our observations remains unknown, this study adds important evidence to support a gain of function hypothesis for the role of hApoEε4 in tau pathology. A large majority of past work has primarily focused on the role of hApoE in Aβ-mediated effects due to the strong correlation between hApoEε4 status and amyloid accumulation in the brain^20^ and there has been a high degree of disappointment surrounding Aβ-reducing therapies in the clinic^83,84^. Thus, therapies to target non-Aβ mediated mechanisms which also underly AD have emerged with a focus on tau. The inhibition of tau oligomerization by the hApoEε4 antibody in this work suggests it may be feasible for an antibody to interfere with hApoEε4-mediated potentiation of tau pathology. Given the critical role of hApoE in the periphery, the ability to identify and leverage CNS-specific mechanisms to engage hApoEε4 would be essential and AAV-mediated delivery of antibodies to distinct cell populations has already been described^85^. AAV-mediated delivery of full-length hApoE antibodies in the brain have been reported to have beneficial effects in preclinical models^86^. Although these studies to date have focused on the ability of hApoE antibodies to decrease Aβ burden^56,57,86^, our work suggests that hApoE represents a unique therapeutic target with the potential to mitigate both Aβ and tau burden in AD.

## Methods

### Rat primary cultures

Primary hippocampal cell cultures were prepared from E18 Sprague–Dawley rat fetuses (BrainBits). Rat hippocampi were trypsinized for 10 min in 0.05% Trypsin–EDTA solution (Invitrogen). Trypsinization was neutralized using DMEM supplemented with 10% heat-inactivated FBS and 10 mM HEPES (all Invitrogen). Cells were dissociated by trituration in plating medium provided by BrainBits containing NBActiv1:Neurobasal/B27/GlutaMax/25 μM glutamate. After 48 h, media was changed over to neuronal maintenance medium (Neurobasal A medium supplemented with B27, 200 mM l-glutamine, and Penicillin Streptomycin; all Invitrogen). Cultures were maintained at 37 °C in a 5% CO2/95% room air-humidified incubator. The maintenance of hippocampal cell cultures was carried out by replacing half of the media every three to four days. For immunocytochemistry, cells were plated into 96-well PDL pre-coated plates (Greiner) at a density of 20,000 cells/well. For biochemistry, cells were plated into 6-well PDL pre-coated plates (Corning) at a density of 1 million cells/well.

### Generation of AAV

Human ApoEε2, ApoEε3, ApoEε4 and codon-optimized wild type human tau (4R2N) were synthesized at Genewiz Inc. A flag tag and HA tag were included at the C-termini of human ApoE and tau constructs respectively during gene synthesis. Synthesized human ApoEε2, ApoEε3 or ApoEε4 were subcloned into a backbone pAAV cis plasmid that contains a human glial fibrillary acidic protein (GFAP) promoter, and HA-tagged human tau was subcloned into a backbone pAAV cis plasmid that contains a human synapsin (SYN) promoter. AAV vectors expressing human ApoEε2, ApoEε3, ApoEε4 and human tau were produced by the helper-free triple-plasmid transfection method at UMASS Gene Therapy Center Vector Core under a fee-for-service agreement. AAV empty vector that only contains the GFAP promoter was generated as a negative control for human ApoEε2-, ApoEε3-, ApoEε4- expressing AAVs and AAV empty vector that only contains the SYN promoter was generated as a negative control for human tau-expressing AAV. The AAV cis plasmid, an adenoviral helper plasmid and a packaging plasmid containing the AAV2 Rep gene and AAV9 Cap gene were co-transfected into HEK293 cells. AAV vectors were subsequently purified by two rounds of cesium chloride density gradient ultracentrifugation. The titers of AAV vectors were determined via RT-PCR analysis and the purity of the AAV preps were determined by silver staining.

### AAV transduction

Cultures were transduced with astrocyte-targeting AAV9’s at DIV5 at a MOI of 20,000 genome copies (GC) per cell (GC/cell). Neuron-targeting AAV9’s were added to cultures at DIV10 at a MOI of 20,000 GC/cell. 50% of the media was removed and replenished 48 h after AAV transduction.

### Immunocytochemistry

Cells were plated in 96-well plates (Greiner) at the optimal density for the both long-term survival of the cells and for the accuracy of high-content image analysis. At the relevant time point following AAV transduction, media was removed, and cells were washed briefly in Dulbecco’s PBS (DPBS) (Sigma). Then, the cells were fixed in 4% paraformaldehyde (Electronic Microscopy Services) in DPBS containing 4% sucrose (both Sigma) for 15 min at room temperature (RT) followed by three washes with DPBS to remove PFA. Fixed cells were kept in DPBS and stored at 4 °C until subsequent immunocytochemistry steps. Cells were incubated in blocking and permeabilization buffer (0.2% Triton X-100, 2% goat serum, and 0.1% BSA in DPBS; all Sigma) for one hour with gentle shaking at RT. After blocking, cells were washed again in DPBS prior to incubation with primary antibodies diluted in antibody solution (2% goat serum, 0.1% BSA in DPBS) overnight at 4 °C with gentle shaking. The day after, plates were washed three times (5 min each with gentle shaking) in DPBS. The following primary antibodies were used for immunostaining throughout this study: MC1 (from Dr. Peter Davies; 2 μg/mL); PHF1 (from Peter Davies; 2 μg/mL) microtubule-associated protein 2 (MAP2; Novus Biologicals NB300-213; 1:1000), hemagglutinin (HA) tag (Biolegend 901501; 1:1000); and GFAP (Novus, 1:1000). The next day, cells were washed three times (5 min each with gentle shaking) in DPBS. Secondary antibodies (Invitrogen A11029, A21245, or A10680) diluted in antibody solution (1:1000) were incubated for 1 h at RT, and subsequently washed three times (5 min each with gentle shaking) in DPBS before image acquisition. Hoechst 33342 (Anaspec) solution was used to stain nuclei during secondary antibody staining (1 μg/mL final concentration). High-content imaging was performed to acquire images using an ArrayScan (Thermo Fisher) with 20X objective before high-content imaging analysis.

### Image acquisition and analysis

To cover a good portion of the surface of the well, ≥ 9 fields per well were imaged, analyzed, and averaged. A total of 3–6 wells of a 96-well plate were used per group (*n* = 3–6) and each experiment was repeated a minimum of three independent times to ensure reproducibility. Data from one representative experiment are shown in each figure. Image analyses and calculations were performed using Cellomics software (ThermoFisher). Hoechst staining was used to label cell nuclei. Single-cell identification was performed using the Hoechst nuclei stain, and the neuronal body and neurites were measured based on the MAP2 stain. Within the cell mask per each experiment, we quantified the MC1- or PHF1-positive total area and total intensity within the MAP2-labeled neurites or cell bodies. Positive areas and intensities were determined positive if they presented a fluorescence intensity of markers staining higher than a defined threshold. The threshold was established based on the distribution of the fluorescence intensity measured in EV or control-treated cells. Defined thresholds were calibrated and standardized the analyses also based on the background fluorescence. For all high-content imaging experiments, both area and intensities of signals were calculated and resulted in similar outcomes. Thus, we only depict the intensity data for several experiments.

### Immunoprecipitation of tau for pTau studies

Cells were rinsed two times in ice-cold PBS and lysates were collected in NP40 buffer (Thermo Fisher) supplemented with protease and phosphatase inhibitor (Thermo Fisher). Protein concentrations were measured using BCA assay (Thermo Fisher) and determined using a standard curve of bovine serum albumin (BSA) serially diluted in PBS. Luminescence was measured on SpectraMax. Equivalent amounts of total protein were added to 50 μL of suspended Protein G Dynabeads (Invitrogen) which were previously pre-cleared and incubated with 4 μg of HA antibody (Biolegend). Lysate was incubated with beads overnight at 4 ˚C. The next day, bead and lysates were washed three times in PBS supplemented with 0.1% Tween-20 (Bio-Rad). 40 μL of elution buffer (Invitrogen) and 1× LDS loading buffer (Invitrogen) was added and samples were boiled at 70 ˚C for 10 min for elute proteins bound to the bead-antibody complex. Samples were then placed on the magnet (Invitrogen) to remove beads and load protein sample using standard Western blot procedures under reducing conditions.

### Lysis of mouse tissues

Tissues were collected and fresh frozen at the time of necropsy. Samples were homogenized at 10% weight/volume in RIPA buffer (Thermo Fisher), supplemented with Halt protease and phosphatase inhibitor cocktail (Thermo Fisher). Samples remained on ice for 30 min for lysis and were homogenized using until complete (Tissue Lyser, Qiagen). Samples were sonicated and spun at 13,000 rpm at 4 ˚C for 10 min to remove debris. Supernatant was collected, aliquoted, and stored at – 80 ˚C until analysis by standard Western blot procedures under reducing conditions.

### Western blot procedure

Protein concentrations of total lysates or conditioned media were measured using BCA assay (Thermo Fisher) and determined using a standard curve of BSA. Conditioned media was pre-clarified using a short low-speed spin (3000×*g*, 10 min) to pellet and remove any cellular debris. Luminescence was measured on SpectraMax. Equivalent amounts of protein were supplemented with final concentrations of 1× LDS sample loading buffer (Life Technologies) and 1× reducing agent (Life Technologies). Samples were boiled at 70 ˚C for 10 min and loaded into appropriate well of 4–12% NuPAGE Bis–Tris gels (Novex, Life Technologies) and standard Western blot procedure was followed. The following antibodies were used for biochemical experiments diluted in Odyssey PBS blocking buffer (LiCor) supplemented with 0.05% Tween-20 (Bio-Rad) after one hour of blocking in room temperature: total tau (Dako A0024; 1:2000); hyperphosphorylated tau (AT8 (Thermo Fisher MN1020, 1:500); PHF1 (from Peter Davies, 1:1000); HA-tagged tau (HA) (Biolegend 901501, 1:1000); flag-tagged hApoE (flag) (Sigma F7425, 1:1000); hApoEε4 9D11 (Biolegend 813701, 1:500); pan-hApoE (Meridian K74180, 1:500); tau-3R (Millipore 05-803; 1:1000); tau-4R (Millipore 05-804; 1:1000); and βIII-tubulin (EMD Millipore AB9354, 1:2000). After overnight incubation with shaking at 4 ˚C in primary antibody of interest, secondary infrared antibodies (LiCor) were added at a concentration of 1:10,000. Odyssey Infrared Imaging System (Li-Cor) and Odyssey software V2.1 was used to scan the immunoblots and analyze the band densities.

### Tau oligomer formation

Recombinant human 4R2N T40 was expressed in bacterial cultures and purified as previously described^40^. Aliquots of recombinant tau were snap-frozen and stored at − 80 °C. To prepare tau oligomers, 5 μM recombinant tau, dissolved in 100 mM MES buffer (4-morpholineethanesulfonic acid hydrate) at pH 6.5, was mixed with 10 μM DTT (BioShop) and incubated for 10 min at 55 °C. Subsequently, 5 μM heparin (Fisher, H19) was added to the solution to induce aggregation and incubated with shaking (1000 rpm) for 4 h at 37 °C. Tau monomers, used as a control in this study, were prepared through an identical protocol without the addition of heparin. This preparation was previously characterized extensively and shown to produce a heterogenous and multimeric population of tau oligomers containing monomers, dimers, trimers, and tetramers^40^.

### Tau oligomer treatment

Tau monomers and tau oligomers were generated as described above. 100 nM of each species was separately added to co-cultures at the timepoint of interest. Cells were washed one day later three times in warmed media. Fresh media was added to the wells and the cells were later processed for immunocytochemistry and high-content imaging at the subsequent timepoint of interest.

### Cholesterol accumulation assay

NBD Cholesterol, a fluorescently-tagged cholesterol, was used as a probe for the detection of cholesterol taken up by cultured cells and used as described by the manufacturer (Cayman Chemicals). Cells were treated at the relevant timepoint in culture with 20 µg/mL (final concentration) of NBD cholesterol and incubated at 37 °C with 5% CO_2_. NBD cholesterol was washed off three times in warmed rat hippocampal maintenance medium four hours after its addition to the media and fresh media was added. Twenty-four hours later, a green background suppressor (Life Technologies) was added to the media and cells underwent live-cell imaging in the presence of a Hoechst 33342 stain (Life Technologies, final concentration of 1 µg/mL) to label nuclei. High-content imaging at 20× magnification and analysis was used to quantify cell number and the intensity of cholesterol associated with nuclei. The background suppressor dye (Life Technologies; following manufacturer’s protocol) was used to prevent the emission of extracellular fluorescence.

### Tau internalization assay

Tau monomers were conjugated to Alexa Fluor 555 (cy3) with a final degree of labeling of 3.2 (3.2 mol of dye/mole of protein) (Life Technologies, Outsourcing Facilities). Cy3-tagged oligomers were produced from cy3-tagged monomers using the *Tau oligomer formation* protocol described above. For internalization assays, cells were treated with 100 nM labeled tau monomers or oligomers (at 37 °C, 5% CO2) in medium. After four hours of incubation with the labeled tau species, cells were rinsed three times in warmed medium. One day later, a Hoechst 33342 stain (Life Technologies; final concentration of 1 μg/mL) was added to label nuclei in the presence of a red background suppressor (Life Technologies). Live-cell imaging was performed under a 20× magnification. High-content imaging and analysis was then used to quantify cy3-positive cells for a set threshold of cy3 fluorescence. The presence of the background suppressor dye (Life Technologies; using manufacturer’s protocol) in the media was to prevent any extracellular fluorescence^40^. To test the effect of heparinase III (Sigma-Aldrich H8891) on tau uptake, co-cultures were treated with the 150 mU/ml enzyme for 4 h prior to the addition of cy3-tau oligomers.

### AlphaLISA to measure total tau and human tau

Cells were plated at density of 30,000 cells/well in a Greiner 96-well PDL pre-coated plate. Seven days after AAV transduction with wild type tau, cells were lysed in 50 μL PhosphoSafe Extraction Reagent (Novagen), supplemented with phosphatase and protease inhibitor cocktail (Thermo Fisher), and centrifuged at 15,000×*g* for 15 min at 4 °C.

For human tau measurement, the commercially available human tau specific AlphaLISA kit was used according to manufacturer’s protocol (Perkin Elmer). Briefly, five microliters of cell lysates were incubated for 1 h at RT with a final concentration of 10 μg/mL tau-acceptor beads and 1 nM biotinylated tau antibody in a 384-well OptiPlate (PerkinElmer). AlphaLISA immunoassay buffer (PerkinElmer) was used as dilution buffer. Diluted AlphaScreen streptavidin coated-donor beads (5 μg/mL final concentration) were added and incubated for 1 h at RT with gentle shaking. The final total reaction volume was 50 µL per well. Luminescence signal was measured using Envision plate reader (Perkin Elmer).

For total (rodent + human) tau measurement, five microliters of cell lysates were incubated for 1 h at RT with a final concentration of 20 μg/mL of BT2-acceptor beads and 0.125 µg/mL biotinylated HT7 antibody in a 384-well OptiPlate (PerkinElmer). AlphaLISA immunoassay buffer (PerkinElmer) was used as dilution buffer. 40 μL of diluted AlphaScreen streptavidin coated-donor beads (final concentration of 20 μg/mL) were added and incubated for 1 h at RT with gentle shaking. The final total reaction volume was 50 µL. Luminescence signal was measured using Envision plate reader (Perkin Elmer).

For both tau assays, all samples were run in duplicate or triplicate and the sample means were used. A minimum of three sample means of technical replicates were used in each experiment.

### AlphaLISA to measure oligomerization of HA-tagged tau

Cells and lysates were processed as described above for other AlphaLISA measurements. Five microliters of cell lysates were incubated for 2 h at RT with a final concentration of 20 μg/mL of HA-acceptor beads in a 384-well OptiPlate (PerkinElmer). AlphaLISA immunoassay buffer (PerkinElmer) was used as a dilution buffer. Diluted AlphaScreen streptavidin coated HA- donor beads at a final concentration of 20 µg/mL were then added and incubated for 1 h at RT with gentle shaking. The final total reaction volume was 50 µL. Luminescence signal was measured using Envision plate reader (Perkin Elmer). All samples were run in duplicate or triplicate and the sample means were used. A minimum of three sample means were used in each experiment.

### Mouse studies

All experiments were performed in accordance with relevant guidelines from our Institutional Animal Care and Use Committee (IACUC) with pre-approved animal protocols, at Merck & Co., Inc., West Point, PA, USA., which were also maintained in accordance to all IACUC protocols.

Tau P301L (“WT/”CAR” rTg4510, which contain a leaky ~ 3-fold overexpression of tau due to the absence of the transactivator) used in these studies were previously described^41^. At eight weeks of age, mice were anaesthetized and underwent intracranial infusion with either 1 µL of PBS control (“sham”) or with 1 µL of AAV9 containing the P_GFAP_::ApoE-Flag construct of interest (1e^10^ GC/site). Hamilton Neuros syringes (2 µL) were used to deliver the intracranial infusion at a flow rate of 0.2 µL/min for 5 min with a 2-min hold before and 5-min hold after infusion of the test material. Stereotactic coordinates used to target the dorsal hippocampus were as follows (mm from Bregma): − 2.5 from AP; + /− 2.00 from ML, − 1.9 from DV. Mice recovered for 72 h before being housed for an additional eight weeks following surgery. At that time, mice were euthanized and tissue was flash frozen with the cerebellum, prefrontal cortex, and left and right hippocampi dissected for subsequent analyses (*n* = 4–6/group). Tissues were stored at – 80 ˚C until the time of analysis.

### Statistics

All data are expressed as mean ± SEM. Statistical analyses were performed using Student's *t* test for comparison between two groups or one-way ANOVA followed by Tukey post hoc test or Fisher’s LSD test for multiple comparisons. Kruskal–Wallis nonparametric test was used when appropriate in substitution for the one-way ANOVA. GraphPad PRISM (7.0) software was used to perform all statistical analyses.

### Consent for publication

Consent for all data from co-authors was received prior to manuscript submission.

## Supplementary Information

Supplementary Figures.

## Data Availability

The datasets used and/or analyzed during the current study are available from the corresponding authors upon reasonable request. All data generated or analyzed during this study are included in this published article.
