# Supplementary material for: Astrocytic expression of the Alzheimer’s disease risk allele, ApoEε4, potentiates neuronal tau pathology in multiple preclinical models
Source: Sci Rep. 2021 Feb 9;11:3438. doi: 10.1038/s41598-021-82901-1 (PMC7873246; doi:10.1038/s41598-021-82901-1)
Supplement: Supplementary file 1 — Supplementary Figures. [file 41598_2021_82901_MOESM1_ESM.docx]

Supplementary Information

Scientific Reports

***Astrocytic expression of the Alzheimer’s disease risk allele, ApoEε4, potentiates***

***neuronal tau pathology in multiple preclinical models***

Angela Marie Jablonski^a*^, Lee Warren^a^, Marija Usenovic^a^, Heather Zhou^b^, Jonathan Sugam^a^,

Sophie Parmentier-Batteur^a^, Bhavya Voleti^a*^

*^a^Neuroscience, MRL, Merck & Co., Inc, 770 Sumneytown Pike, West Point, PA, USA 19486*

*^b^Genetics and Pharmacogenomics, MRL, Merck & Co., Inc, 2000 Galloping Hill Rd, Kenilworth,*

*NJ, USA 07033*

*Correspondence can be addressed to A.M.J. (angela.jablonski@merck.com) or B.V. (bhavya.voleti@merck.com).

This Supplementary Information contains four figures.


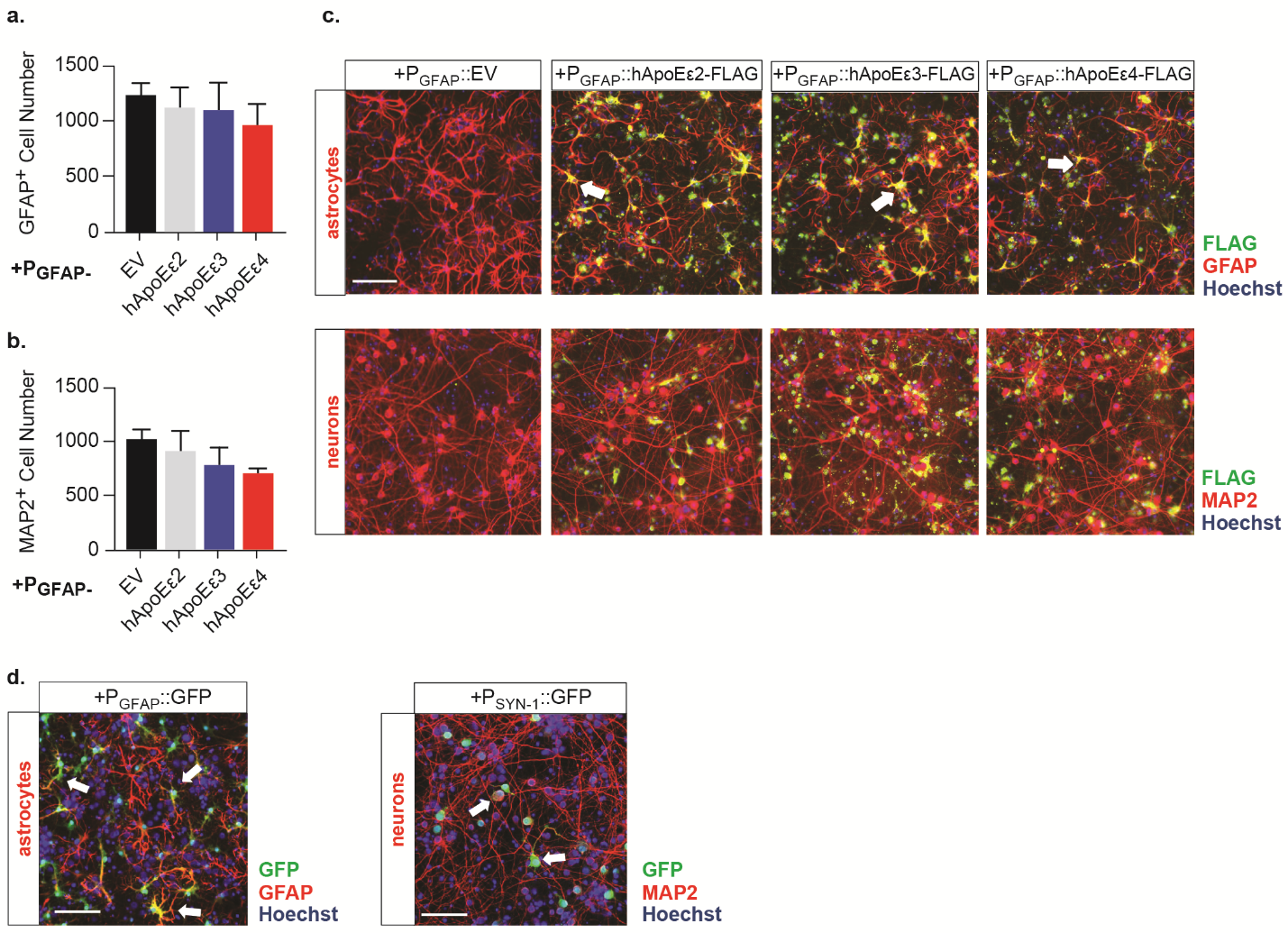


**Supplementary Figure S1 | Localization of human ApoE constructs under control of GFAP promoter.** Data corresponds to one representative experiment. (a-b) Quantification of GFAP-positive (panel a) and MAP2-positive (panel b) nuclei stained with Hoechst. Data reflect immunocytochemistry visualized in panel c (*n* = 3 wells / group). (c) Scale bar represents 50µm. Immunocytochemistry (20X magnification) to detect flag-tagged hApoE construct within astrocytic (top panel; GFAP-positive) or neuronal (bottom panel; MAP2-positive) cell populations. Rat hippocampal co-cultures were transduced with flag-tagged hApoE construct or empty vector under the GFAP promoter at DIV5 and fixed and stained five days later. Arrowheads highlight flag tag detection within GFAP-labeled astrocytes. (d) Immunocytochemistry to detect the expression of GFP driven under the control of astrocytic (left panel) or neuronal (right panel) promoters. Rat hippocampal co-cultures were co-stained for astrocytic (GFAP) or neuronal (MAP2) markers respectively. Arrowheads highlight GFP expression in respective cell types.


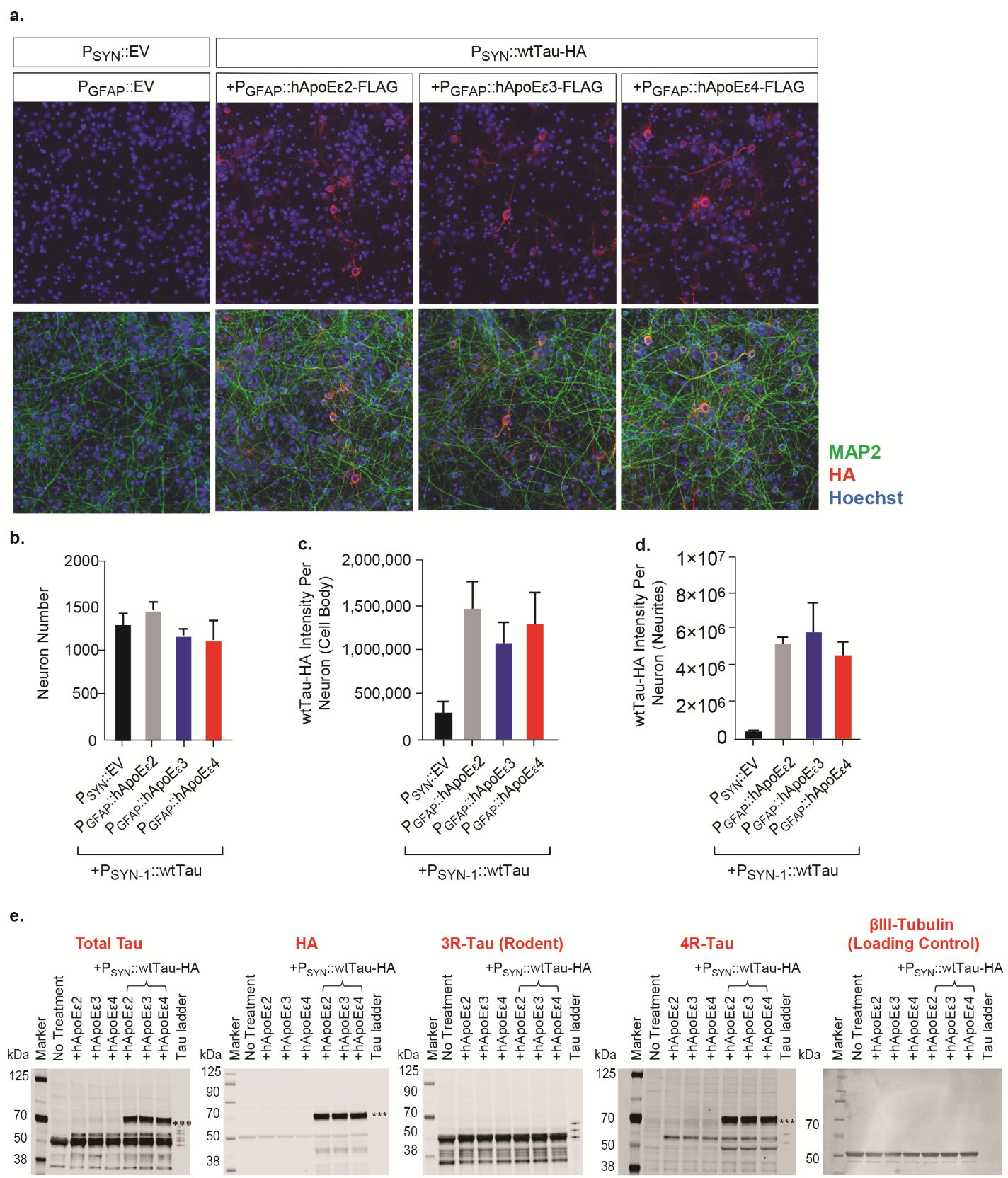


**Supplementary Figure S2 | wtTau-HA construct expression is not increased by hApoEε4 overexpression.** Data corresponds to one representative experiment. (a) Immunocytochemistry (20X magnification) to detect expression of HA-tagged tau construct in rat hippocampal co-cultures alongside transduction with hApoE isoforms or empty vector control (DIV17). (b-d) Quantification of high-content imaging of immunocytochemistry depicted in panel a (*n* = 3 wells / group). (b) Number of MAP2-positive nuclei for immunocytochemistry depicted in panel a (*p* = 0.2204, one-way ANOVA). (c-d) Quantification of HA intensity normalized per neuron in the cell bodies (c) (*p* = 0.6607, one-way ANOVA) and neurites (d) (*p* = 0.6738, one-way ANOVA) of MAP2-labeled neurons visualized in panel a. (e) Western blots of total cell lysates prepared from rat hippocampal co-culture transduced with hApoE constructs with and without human tau (4R2N) overexpression. Lysates were made in Phosphosafe buffer (Millipore) supplemented with protease and phosphatase inhibitor (Thermo Fisher) before being probed with the following antibodies: total tau to detect all tau species; HA to detect the transduced HA-tagged tau construct; 3R-tau to detect the endogenous rodent tau;, and 4R-tau to primarily detect human tau construct. β-tubulin was used as loading control. Asterisks (***) highlight the band corresponding to the introduced HA-tagged human tau construct.


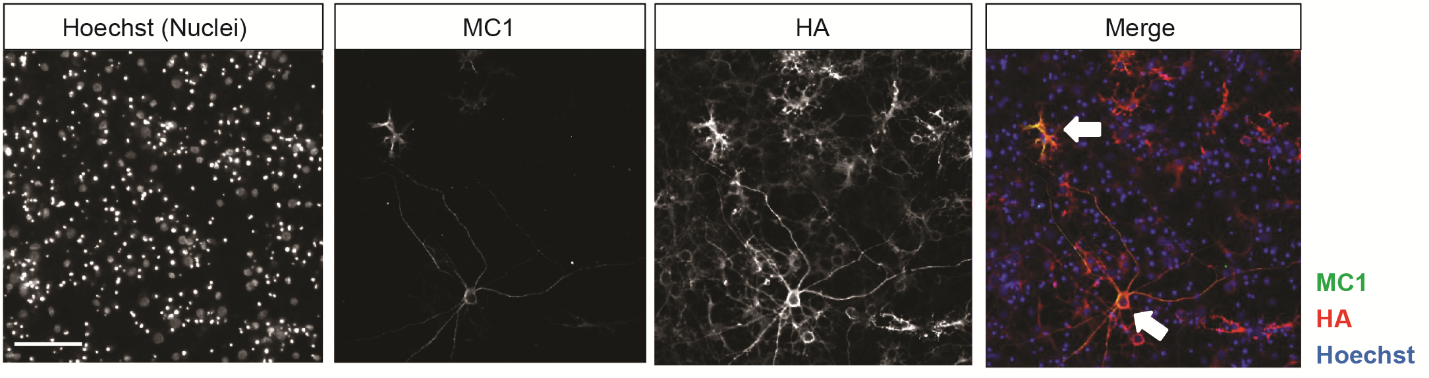


**Supplementary Figure S3 | Not all tau-HA positive cells contain MC1-positive tau pathology.** Scale bar represents 50µm. Representative immunocytochemistry (20X magnification) of rat hippocampal co-cultures stained for MC1 and HA against Hoechst nuclei stain. Cells were fixed and stained seven days (DIV17) following transduction with the wtTau-HA construct. Arrows highlight cells which are positive for both MC1 and HA.

**
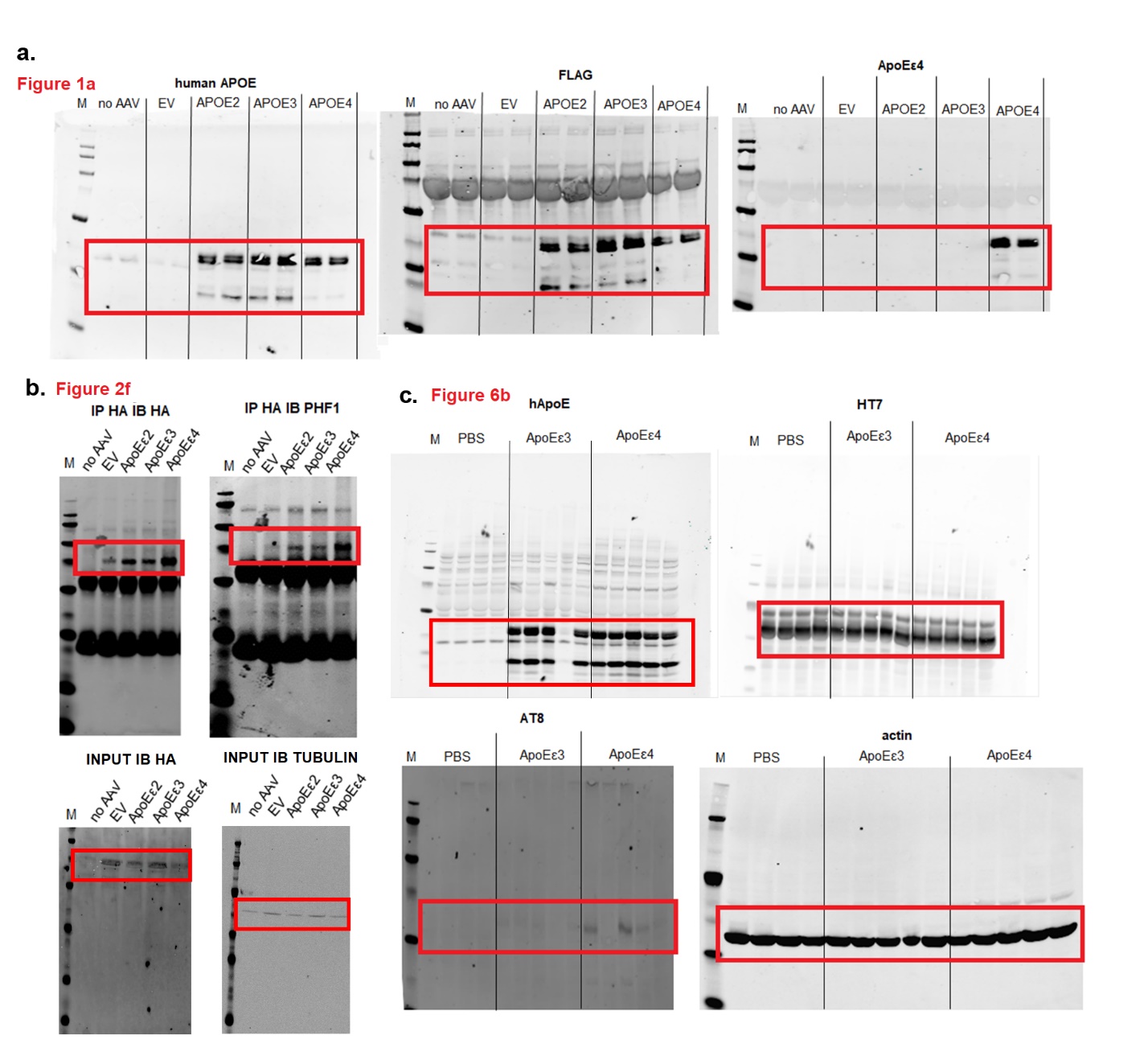
**

**Supplementary Figure S4 | Western blot images corresponding to cropped figures in the main text.** Uncropped Western blot images of data used throughout the main text of manuscript. (a) Western blot images corresponding to blots presented in Figure 1a. (b) Western blot images corresponding to blots presented in Figure 2f. (c) Western blot images corresponding to blots presented in Figure 6b.
